# Evolution of the exclusively human pathogen *Neisseria gonorrhoeae*: Human‐specific engagement of immunoregulatory Siglecs

**DOI:** 10.1111/eva.12744

**Published:** 2019-01-03

**Authors:** Corinna S. Landig, Ashley Hazel, Benjamin P. Kellman, Jerry J. Fong, Flavio Schwarz, Sarika Agarwal, Nissi Varki, Paola Massari, Nathan E. Lewis, Sanjay Ram, Ajit Varki

**Affiliations:** ^1^ Glycobiology Research and Training Center University of California, San Diego La Jolla California; ^2^ Department of Cellular and Molecular Medicine University of California, San Diego La Jolla California; ^3^ Department of Medicine University of California, San Diego La Jolla California; ^4^ Department of Earth System Science Stanford University Stanford California; ^5^ Department of Pediatrics University of California, San Diego La Jolla California; ^6^ Bioinformatics and Systems Biology Graduate Program University of California, San Diego La Jolla California; ^7^ Department of Medicine University of Massachusetts Medical School Worcester Massachusetts; ^8^ Department of Pathology University of California, San Diego La Jolla California; ^9^ Department of Immunology Tufts University School of Medicine Boston Massachusetts; ^10^ Novo Nordisk Foundation Center for Biosustainability University of California, San Diego La Jolla California

**Keywords:** disease biology, evolutionary medicine, gonorrhea, microbial biology, polymorphism, population genetics, sialic acid, Siglecs

## Abstract

*Neisseria gonorrhoeae* causes the sexually transmitted disease gonorrhea exclusively in humans and uses multiple strategies to infect, including acquisition of host sialic acids that cap and mask lipooligosaccharide termini, while restricting complement activation. We hypothesized that gonococci selectively target human anti‐inflammatory sialic acid‐recognizing Siglec receptors on innate immune cells to blunt host responses and that pro‐inflammatory Siglecs and *SIGLEC* pseudogene polymorphisms represent host evolutionary adaptations to counteract this interaction. *N. gonorrhoeae* can indeed engage multiple human but not chimpanzee CD33rSiglecs expressed on innate immune cells and in the genitourinary tract––including Siglec‐11 (inhibitory) and Siglec‐16 (activating), which we detected for the first time on human cervical epithelium. Surprisingly, in addition to LOS sialic acid, we found that gonococcal porin (PorB) mediated binding to multiple Siglecs. PorB also bound preferentially to human Siglecs and not chimpanzee orthologs, modulating host immune reactions in a human‐specific manner. Lastly, we studied the distribution of null *SIGLEC *polymorphisms in a Namibian cohort with a high prevalence of gonorrhea and found that uninfected women preferentially harbor functional *SIGLEC16* alleles encoding an activating immune receptor. These results contribute to the understanding of the human specificity of *N. gonorrhoeae *and how it evolved to evade the human immune defense.

## 1 INTRODUCTION

Gonorrhea is a sexually transmitted disease that poses a major global health problem, with about 78 million estimated infections worldwide in 2012 (Newman et al., 2015). This disease is caused by *Neisseria gonorrhoeae*, a Gram‐negative bacterium that exclusively infects humans. Urogenital epithelia are the main sites of infection, but *N. gonorrhoeae* can also infect the conjunctiva, pharynx, and rectal mucosa (Edwards & Apicella, 2004). Gonococci successfully proliferate in different host microenvironments and evade the human immune system by constantly modulating their surface antigenic makeup by phase variation and other mechanisms (Criss & Seifert, 2012; Edwards & Apicella, 2004; Virji, 2009).

Dynamic changes in the glycan extensions of gonococcal lipooligosaccharide (LOS) are an excellent example of how *N. gonorrhoeae* evade the host immune response. The lacto‐*N*‐neotetraose (LNnT) structure of the LOS engages host surface receptors, like the asialoglycoprotein receptor (ASGP‐R), and leads to the invasion of the urethral epithelia (Harvey, Jennings, Campbell, Williams, & Apicella, 2001). Sialic acid, a 9‐carbon backbone acidic sugar, plays an important role during the infection of many human‐specific pathogens (Hentrich et al., 2016; Patrone & Stein, 2007; Varki & Gagneux, 2012). The gonococcal LNnT structure becomes sialylated by the gonococcal sialyltransferase (Lst), a surface‐exposed outer membrane protein that uses host sialic acid in form of CMP‐Neu5Ac (Shell, Chiles, Judd, Seal, & Rest, 2002). Sialylation helps the bacteria recruit factor H to become more resistant to complement‐mediated killing (Ram et al., 1998, 1999) and blocks antibody recognition of select underlying structures (Elkins et al., 1992).

Sialic acids on mammalian cell surfaces are also recognized as “Self‐Associated Molecular Patterns” (SAMPs) by other receptors of the host immune system (Varki, 2011). Interactions of sialylated host glycans with inhibitory members of the sialic acid‐binding immunoglobulin superfamily lectins (Siglecs) maintain immune cells in a quiescent state and dampen unwanted inflammation (Crocker, Paulson, & Varki, 2007; Varki, 2011; von Gunten & Bochner, 2008). Inhibitory effects are driven by ITIM motifs in Siglec intracellular domains, which recruit SHP phosphatases and quench pro‐inflammatory signaling cascades. This property is subverted by pathogens to control host immune responses and escape elimination. For example, group B streptococci (GBS) evade host immune responses by engaging inhibitory Siglec‐5 and Siglec‐9 (Carlin, Lewis, Varki, & Nizet, 2007).

However, humans also express paired Siglecs––sets of receptors with similar ligand specificities but opposite signaling properties. For instance, the Siglec‐5/‐14 and Siglec‐11/‐16 pairs have a highly conserved extracellular domain and similar ligand specificities (Angata, Hayakawa, Yamanaka, Varki, & Nakamura, 2006; Cao et al., 2008; Schwarz, Fong, & Varki, 2015; Wang, Mitra, Cruz, et al., 2012). Activating Siglecs recruit adaptor proteins such as DAP10 and DAP12 through a positively charged amino acid in the transmembrane domain (Angata et al., 2006; Cao et al., 2008; Pillai, Netravali, Cariappa, & Mattoo, 2012; Tourdot et al., 2013). The adaptor proteins contain ITAM motifs that recruit Syk kinase, which induces a pro‐inflammatory signaling cascade (Lanier, 2009). It has been suggested that activating Siglecs represent evolutionary responses to microbes exploiting inhibitory Siglecs. In fact, Siglec‐14 has been shown to counteract the exploitation of Siglec‐5 by GBS (Ali et al., 2014), and Siglec‐16 reduces survival of *E. coli *K1 during infection (Schwarz et al., 2017). Moreover, due to polymorphic pseudogenization, not all humans are able to express these activating Siglec receptors, generating different immune responses to infections (Angata et al., 2013; Schwarz et al., 2015). Notably, in some instances, Siglec‐14 blocking antibody reversed pathogens have also evolved direct interactions of Siglecs with their surface proteins (Carlin et al., 2009). In this study, we elucidate how *N. gonorrhoeae* has evolved both sialic acid‐dependent and sialic acid‐independent interactions with human but not chimpanzee Siglecs to modulate their pathogenic potential and the host inflammatory response in a species‐specific manner, thus contributing to the host restriction of gonorrhea. We also explore the impact of Siglec polymorphisms in a population at high risk of gonorrhea.

## 2 MATERIALS AND METHODS

### 2.1 Bacteria and cell lines

*Neisseria gonorrhoeae* F62 was isolated from an uncomplicated infection (Kellogg, Peacock, Deacon, Brown, & Pirkel, 1963). *Neisseria gonorrhoeae* 15253 was isolated from a disseminated infection (O'Brien, Goldenberg, & Rice, 1983). Both strains were piliated. The mutant strains of F62 (ΔlgtD, ΔlgtA, ΔlgtE, and ΔlgtF) were constructed using plasmids and methods described previously (Gulati et al., 2015). The LOS phenotype of the mutants was verified by LOS staining of protease K‐treated whole‐cell samples that were separated on a 12% Bis–Tris gel with MES running buffer. *N. gonorrhoeae* were grown overnight on chocolate agar plates with IsoVitaleX (BD Bioscience) or in GC broth supplemented with IsoVitaleX at 37°C and 5% CO_2_. When indicated, growth media was supplemented with 30 µM CMP‐Neu5Ac (Nacalai USA, Inc.). Incorporation of Neu5Ac has been confirmed by loss of Erythrina Cristagalli Lectin (ECA, Vector Laboratories), which binds to the lactosamine epitope. For all binding and infection studies, bacteria were cultivated to an optical density at 600 nm equivalent to 0.4–0.6. THP‐1 cells were grown in RPMI‐1640 (Gibco) with 10% fetal calf serum (Gemini Bio‐Products) at 37°C and 5% CO_2_.

### 2.2 Siglec‐Fc production

Siglec‐Fcs were produced as described in Padler‐Karavani et al. (2014). Siglec‐Fc vectors were transfected into HEK293A cells cultured in serum‐free media supplemented with Nutridoma‐SP (Roche). Culture supernatants were collected, and Siglec‐Fcs were purified on a Sepharose Protein A Column (GE Healthcare Life Sciences). After washing with Tris‐buffered saline (20 mM Tris–HCl, 150 mM NaCl, pH 8.0; TBS), Siglec‐Fcs were desialylated on column by neuraminidase from Arthrobacter ureafaciens (Sigma‐Aldrich) for 1 hr at room temperature. After extensive washing with TBS, Siglec‐Fcs were eluted with 0.1 M glycine–HCl pH 3.0 and pH is neutralized immediately. Siglec‐Fcs are concentrated by Amicon centrifugal filters (Millipore). The functionality of Siglec‐Fcs has been pretested on sialoglycan arrays.

### 2.3 Bacteria binding assay

Ninety‐six‐well plates were coated with 1 μg/well protein A (Thermo Scientific) in 50 mM carbonate buffer pH 9.5 overnight at 4°C. Wells were washed with PBS‐T (0.05% Tween‐20 in PBS) and blocked with 1% BSA in PBS for 1 hr at room temperature. 1 μg/well Siglec‐Fcs were incubated for 2 hr at room temperature. To correct for unspecific binding of Fc part, human IgG was incubated in parallel. Afterward, wells were washed with PBS‐T. *N. gonorrhoeae* were pelleted, washed with HBSS, and then incubated with 0.1% fluorescein isothiocyanate (FITC, Sigma) in PBS for 1 hr at 37 ˚C with rotation. Bacteria were extensively washed with HBSS to remove trace amounts of free FITC and then resuspended in HBSS at an optical density of 1 at 600 nm. A volume of 0.1 ml of FITC‐labeled bacteria was added to each well. Plates were centrifuged at 500 g for 10 min and incubated for 1 hr at room temperature. After washing to remove unbound bacteria, the residual fluorescent intensity was measured using a SpectraMax M3 (Molecular Devices). Unspecific binding signal from human IgG was subtracted as background from data. Binding of Siglec‐Fc to *N. gonorrhoeae* was performed by incubating ~10^7^ bacteria with the indicated concentrations of Siglec‐Fc for 30 min at 37°C. Bound Siglec‐Fc was detected with anti‐human IgG FITC on a FACSCalibur, and data were analyzed using FlowJo software.

### 2.4 Binding assays to porins

Ninety‐six‐well plates were coated with 1 μg/well of purified PorinB.1A and PorinB.1B. Purified porins are in their native trimeric state in proteosomes and purified as described in Ref. Massari, King, MacLeod, and Wetzler (2005) or purified IgA‐Br as negative control (Fong et al., 2015) in 50 mM carbonate buffer pH 9.5 overnight at 4°C. Wells were washed with PBS‐T (0.05% Tween‐20 in PBS) and blocked with 1% BSA in PBS for 1 hr at room temperature. Wells were washed and incubated with 5 µg/ml Siglec‐Fc in 1% BSA PBS‐T for 2 hr at room temperature. Wells were washed and incubated with goat HRP‐conjugated anti‐human IgG (1:5,000 dilution, Jackson ImmunoResearch Laboratories Inc.) for 1 hr at room temperature. Wells were washed and incubated with TMB substrate (BD OptEIA), and absorbance was measured using a SpectraMax M3 (Molecular Devices).

### 2.5 Cytokine secretion analysis

THP‐1 cells expressing Siglec‐5 or Siglec‐14 (Yamanaka, Kato, Angata, & Narimatsu, 2009) were differentiated with 10 ng/ml PMA in a 24‐well plate for 24 hr and infected with MOI = 1 of the same inoculum of *N. gonorrhoeae* F62. Supernatant was collected after 24 hr. IL‐6 concentration was measured with ELISA standard kit from BioLegend.

### 2.6 Phagocytosis assay

THP‐1 cells transduced with an expression plasmid for Siglec‐5, Siglec‐14, or empty vector were differentiated with 12.5 ng/ml of phorbol myristate acetate (PMA) for 24 hr. Next, the cells were washed with sterile culture media, blocking or nonblocking antibodies were added, and then exposed to 10 µg/ml of PIB for 10 min before the addition of pHrodo Red *S. aureus* BioParticles (prepared as per manufacturer's instructions, Thermo Fisher Scientific). After incubation at 37°C for 2 hr, the cells were washed with PBS and then detached with 5 mM EDTA in PBS. The detached cells were centrifuged for 5 min at 500 g and washed with PBS. After another centrifugation, the cells were resuspended in PBS and assayed for phagocytosis of the pHrodo BioParticles by flow cytometry. All tests were performed in triplicate.

### 2.7 Immunohistochemistry

Samples used for immunohistochemistry were heterozygous for Siglec‐16 or homozygous null (negative control). All three human cervix samples that were used had both squamous epithelium and columnar epithelium as well as the junctional zone. Samples of human spleen similarly either heterozygous for Siglec‐16 (positive control) or homozygous null (negative control) were used with every experiment. Deparaffinized sections were blocked for endogenous peroxidases and endogenous biotin and subjected to heat‐induced antigen retrieval using pH 9.0 buffer and pressure cooker de‐cloaking chamber (Biocare Medical) for 15 min at 110 degrees. After the slides cooled to 37°C, the sections were overlaid with primary antibodies at the appropriate dilutions and incubated in a humid chamber overnight at 4°C. Following washing after each incubation, the sections were sequentially overlaid with biotinylated anti‐mouse, HRP‐Streptavidin, and biotinyl tyramide to amplify, either HRP‐Streptavidin again or fluorescently labeled streptavidin. If HRP‐Streptavidin was the final step, the substrate used for color development was AEC (Vector laboratories) and nuclei were counterstained with Mayer's hematoxylin and the slides were aqueous mounted for viewing and photography using a Olympus Magnafire digital photomicrography on an Olympus BH2 light microscope. If fluorescence tags were used, the nuclei were counterstained using Hoechst and coverslipped and viewed with photography using the Keyence BZ9000 with the appropriate filters.

### 2.8 Genotyping of Siglecs in Namibian cohort

Genomic DNA was isolated from FTA cards of Namibian cohort (Hazel, Ponnaluri‐Wears, Davis, Low, & Foxman, 2014) with E.Z.N.A. MicroElute Genomic DNA Kit (OMEGA). The participants of the Namibian cohort study were randomly selected and not based on any reported symptoms. Genotyping of the Siglec‐3 polymorphism rs3865444 was performed as described in Schwarz et al. (2016). Genotyping of Siglec‐5 and Siglec‐14 was performed as described in Yamanaka et al. (2009). Genotyping of Siglec‐16 was performed by PCR with primers (forward: GCATGTCTGATCACCTCAGTTGGGAAAG; reverse: CCCTGACTCTCCTGTACTGATAAACC) and OneTaq MasterMix (New England Biolabs). Followed by restriction digest with TspRI (New England Biolabs), which cuts a polymorphism (Wang, Mitra, Secundino, et al., 2012) in disequilibrium with the SIGLEC16P.

### 2.9 Statistical analysis

Prism 6 software (GraphPad) was used for statistical analyses of binding, cytokine, and phagocytosis assays. Quantitative data are expressed as means  ±  standard deviation (*SD*, represented as error bars). Unpaired Student's *t* test or ANOVA was used for comparisons involving two groups.

Gene Association Analysis: Gene–disease association was assessed between binary variables representing each genotype (homozygous dominant, heterozygous, homozygous recessive) of the three loci (Siglec‐3, Siglec‐16, and Siglec‐5/14), and we used models considering the presence or absence of each genotype. Standard genetic models of dominant, recessive, additive, and epistatic were examined but discarded due to overfitting. The unusually high frequency of disease mutations seen in Namibian cohort interfered with the efficacy of common models. Binary genotype variables were used instead. These models are sufficient to assess univariate and bivariate gene–disease associations provided two genotype variables corresponding to one gene are not used in the same model; this results in nonexistent genome models and therefore was avoided.

Models were learned from gender‐segregated data to account for the unique mechanisms of virulence across genders. Univariate logistic regressions were constructed to predict the magnitude and significance of association between each genotype and the disease. The univariate models provided assessments of the single genotype contributions to the disease. Multivariate additive was constructed using the R::MASS::stepAIC function. Multivariate additive models were constructed using backward selection from the complete additive model including all genotypes. Code is deposited at github.com/bkellman/Gh_genotypes/.

## 3 RESULTS

### 3.1 Siglec‐11 and Siglec‐16 are expressed on human cervical epithelium

**Figure 1:**
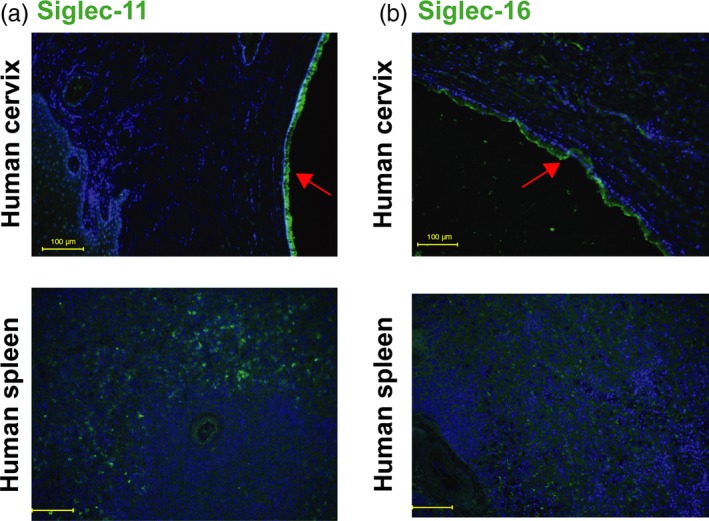
Siglec‐11 and Siglec‐16 are expressed on human cervical epithelium. Immunohistochemical analysis of paraffin sections of human cervix samples, with spleen as positive control using mouse monoclonal. (a) anti‐Siglec‐11 and (b) anti‐Siglec‐16 antibodies (green). Nuclei are blue (Hoechst), red arrows indicate columnar cervical epithelium, and yellow bar indicates 100 µm. Images are representative of *n* = 3. The samples of human Cervix and spleen shown were heterozygous for *SIGLEC16*

Siglecs are mainly found on cells of the innate immune system: For example, monocytes express Siglec‐3 and Siglec‐9; neutrophils express Siglec‐5, Siglec‐9, and Siglec‐14 (Crocker et al., 2007); and macrophages express Siglec‐3, Siglec‐11, and Siglec‐16. Some Siglecs also have been found on other cell types; for example, Siglec‐11 on ovarian fibroblasts (Wang et al., 2011), Siglec‐5 on human but not chimpanzee amniotic epithelia (Ali et al., 2014), and Siglec‐6 on human placental trophoblast, but not in great apes (Brinkman‐Van der Linden et al., 2007). The primary site of gonococcal infection in women is the endocervical epithelium. We detected Siglec‐11 and Siglec‐16 expression on human cervical columnar epithelium (Figure 1), but not Siglec‐3, Siglec‐5, Siglec‐9, and Siglec‐14 (data not shown). Cervical squamous epithelia did not show any Siglec‐11 or Siglec‐16 expression (data not shown). The presence of Siglec‐11 and Siglec‐16 on the cervical columnar epithelium, the main site of gonococcal infection in females, may play a crucial role in host defense. We hypothesize that *N. gonorrhoeae *engages the inhibitory Siglec‐11, which downregulates the pro‐inflammatory response and enables the bacteria to escape immune detection and establish asymptomatic infection. Siglec‐11 is expressed in all humans; however, due to a polymorphism, not all humans are able to express Siglec‐16 (Wang, Mitra, Cruz, et al., 2012). In individuals who express this activating receptor, Siglec‐16 could enable the host to detect *N. gonorrhoeae* and mount a pro‐inflammatory immune response that results in symptoms and facilitates clearance of bacteria.

### 3.2 *Neisseria gonorrhoeae* selectively engages human Siglec receptors

**Figure 2:**
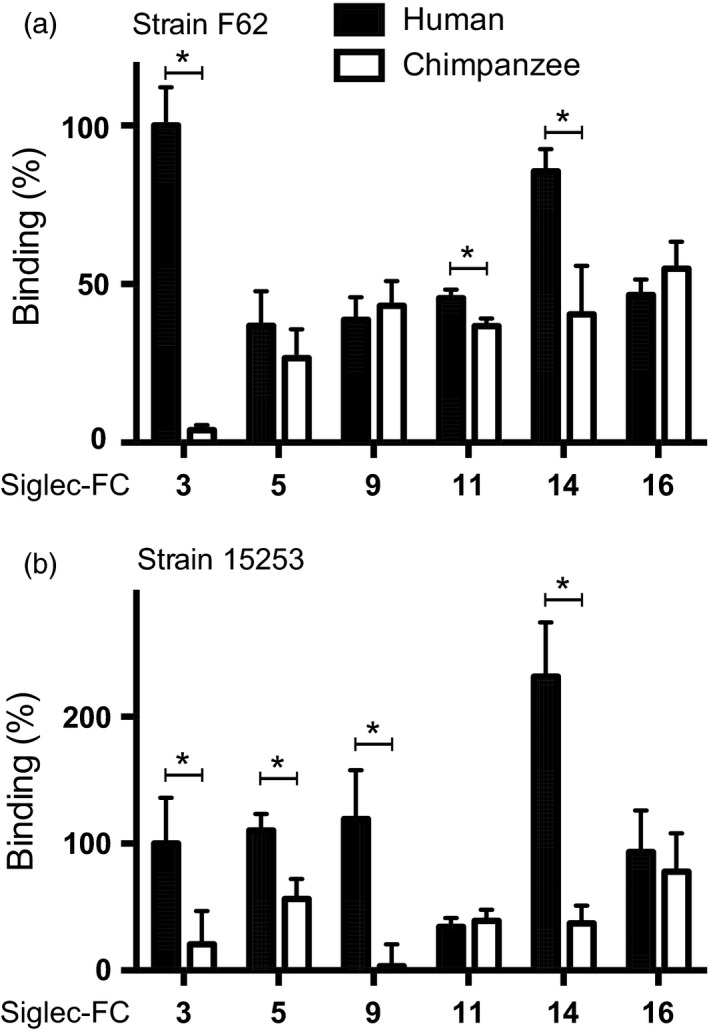
Human Siglecs bind selectively to *Neisseria gonorrhoeae.* Recombinant human and chimpanzee Siglec‐Fc fusion proteins were immobilized, and binding of FITC‐labeled, sialylated *N. gonorrhoeae* (a) strain F62 and (b) strain 15253 was measured. Binding is normalized to human Siglec‐3‐Fc and corrected for unspecific binding to Fc part (huIgG). Data were analyzed with *t* test and represented as mean ± *SD*, *n* = 3

Natural infection with *N. gonorrhoeae *occurs exclusively in humans. Siglecs are widely expressed on cell surfaces of innate immune cells such as macrophages and neutrophils, which produce key innate immune responses to gonococcal infections (Virji, 2009). We focused on Siglecs 3, 5, 9, 11, 14, and 16, which are prominent examples of Siglecs expressed by phagocytes and/or epithelial cells that are relevant to gonococcal infection in humans. To determine whether gonococci interact with Siglecs in a human‐specific manner that may contribute to its human specificity, we compared binding of recombinant soluble extracellular domains of human and chimpanzee homologs (fused with Ig‐Fc) to *N. gonorrhoeae* strains F62 and 15253 (Figure 2). While strain F62 can express the LNnT LOS epitope that can be terminally substituted with sialic acid via an α2‐3 linkage (Yamasaki, Nasholds, Schneider, & Apicella, 1991), strain 15253 lacks the genes that encode LOS glycosyltransferases LgtB, LgtC, and LgtD (Erwin, Haynes, Rice, & Gotschlich, 1996) and thus can expresses LOS structures with only lactose extensions from HepI and HepII (Supporting Information Figure S6a) (Mandrell, Griffiss, Smith, & Cole, 1993). While human Siglec‐3 (also known as CD33) bound strongly to strain F62 (grown in media containing CMP‐Neu5Ac to sialylate LOS, Supporting Information Figure S1), the chimpanzee homolog showed no binding (*p* = 0.002; Figure 2a). F62 bound to all other human and chimpanzee Siglecs tested (Siglecs 5, 9, 11, 14, and 16), with human Siglec‐11 (*p* = 0.013) and Siglec‐14 (*p* = 0.010) also showing a significantly increased binding over the chimpanzee homologs. The *N. gonorrhoeae *PorB.1A strain 15253, which was isolated from a disseminated infection, bound significantly better to human Siglec‐3 (*p* = 0.037), Siglec‐5 (*p* = 0.011), Siglec‐9 (*p* = 0.009), and Siglec‐14 (*p* = 0.002) than to its chimpanzee homolog (Figure 2b). The LOS of strain 15253 present in bacterial Triton X‐100 extracts can be sialylated upon addition of ^3^H‐labeled CMP‐Neu5Ac (Mandrell, Smith, Jarvis, McLeod, & Cole, 1993). We recently confirmed the addition of Neu5Ac to the lactose extension from HepII on intact bacteria when strain 15253 was grown in the presence of CMP‐Neu5Ac (Ram et al., 2018).

**Figure 3:**
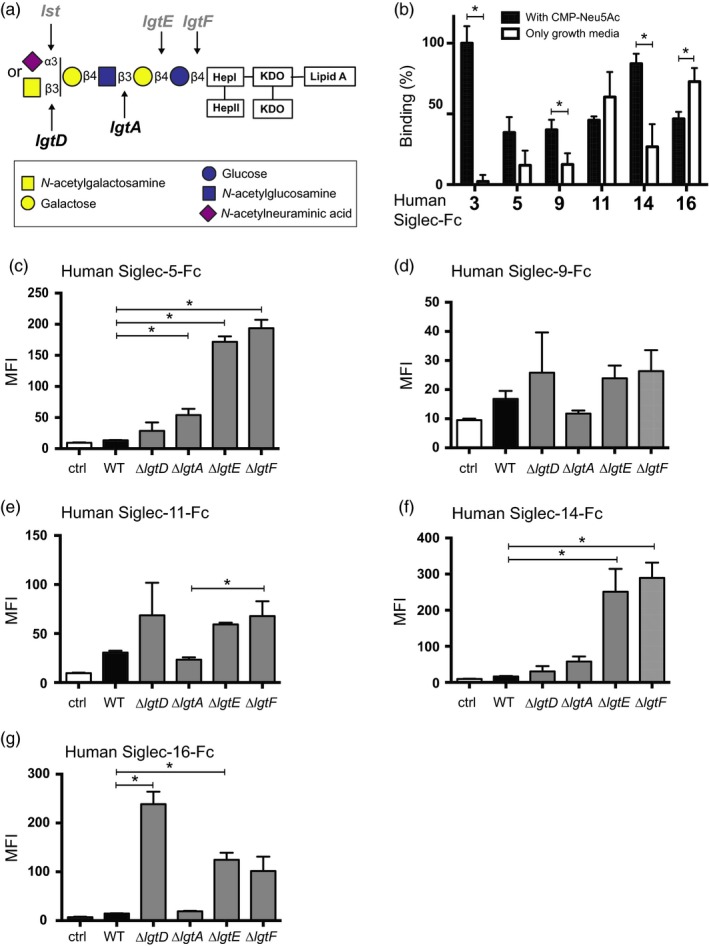
Siglec interactions with gonococci are sialic acid‐dependent and sialic acid‐independent. The LNnT LOS (a) of *N. gonorrhoeae *F62 strain can be sialylated by the sialyltransferase (Lst) in the presence of CMP‐Neu5Ac. Phase‐variable genes are presented in gray. (b) Binding FITC‐labeled F62 grown with and without CMP‐Neu5Ac to immobilized human Siglec‐Fcs. Binding is normalized to human Siglec‐3‐Fc and corrected for unspecific binding to Fc part (huIgG). Data were analyzed with *t* test and represented as mean ± SD, *n* = 3. (c–g) Binding of human Siglec‐Fcs to F62 mutants where genes that encoded the glycosyltransferases *lgtD*, *lgtA*, *lgtE,* or *lgtF* were deleted, which lead to progressive truncation of the LOS, was measured by flow cytometry. Bacteria (~10^7^ CFU) were incubated with 10 µg/ml of each Siglec‐Fc for 15 min at 37°C and surface‐bound Siglec‐Fcs were detected with anti‐human IgG (Fc specific) conjugated to FITC. Controls represent bacteria incubated with anti‐human IgG FITC alone (no added Siglec‐Fc). Data are represented as mean ± *SEM*, *n* = 3

Amino acid sequence differences, as well as differences in post‐translational modifications such as *N*‐ and O‐glycosylation between the human and chimpanzee homologs, could be responsible for the difference in binding (Supporting Information Figures [Link], [Link], [Link], [Link]). Differences in patterns of Siglec binding between the strains may also be due to differences in the LOS structure (Figure 3a), including sialylation, and/or other surface structures that could modify interactions.

### 3.3 *Neisseria gonorrhoeae* binding to Siglecs is only partially dependent on LOS sialic acid, and truncation of LOS increases binding

Since Siglecs are lectins that usually bind to sialic acid, we hypothesized that the sialylated LOS on *N. gonorrhoeae *(Figure 3a) is likely the primary ligand for Siglecs. Growth of *N. gonorrhoeae* in media lacking CMP‐Neu5Ac leads to expression of non‐sialylated LOS because gonococci lack the ability to synthesize sialic acid de novo*; *the addition of CMP‐Neu5Ac to media results in sialylation of LNnT LOS (Mandrell, Smith, et al., 1993; Nairn et al., 1988; Supporting Information Figure S1). The binding of sialylated and non‐sialylated gonococci F62 indeed differs with some Siglecs (Figure 3b). The binding of Siglec‐3 (*p* < 0.001) is solely dependent on sialic acid. The binding of Siglec‐9 (*p* = 0.016) and Siglec‐14 (*p* = 0.004) was enhanced by sialic acid. However, residual binding suggested the presence of sialic acid‐independent ligands. In contrast, the binding of Siglec‐16 to F62 was significantly increased (*p* = 0.013) in the absence of sialic acid. Similar results were observed with gonococcal strain 15253, with the exception of Siglec‐16, where sialylation of 15253 did not change the binding (Supporting Information Figure S6b). Together with the finding in Figures 1 and 2, these results support the hypothesis that structures in addition to sialic acids can mediate the interactions of Siglecs and gonococci. It is possible that the glycan‐binding pocket that confers lectin‐like properties to Siglecs also binds to other glycan structures. To elucidate whether Siglecs bind to non‐sialylated LOS structures, we tested Siglec binding by flow cytometry to isogenic mutants of strain F62 that expressed truncated LOS glycans by (separately) inactivating the LOS glycosyltransferases *lgtD, lgtA, lgtE, *and *lgtF* (Figure 3a). Progressive truncation of LOS correlated with increased binding of Siglec‐5 and Siglec‐14 to F62 (Figure 3c,f). This inverse correlation between Siglec binding and LOS glycan length was not significant for Siglec‐9 (Figure 3d). The binding of Siglec‐11‐Fc increases significantly with the truncation of the lactose epitope (Figure 3e). Siglec‐16‐Fc binds the strongest to the mutant with the exposed (unsialylated) LNnT structure (Figure 3g). These data suggest the presence of an alternate ligand on *N. gonorrhoeae* for Siglecs. When present, sialylated LOS may be the primary binding site for LOS. In contrast, as with Siglec‐11 and Siglec‐16, sialylated LNnT LOS may actually hinder access of the alternate ligand to Siglecs. However, when LOS phase varies (e.g., lgtA “off”), it may leave the alternate ligand more exposed and permit binding to Siglecs.

### 3.4 Gonococcal Porin B mediates binding to human Siglecs in a protein‐dependent manner

**Figure 4:**
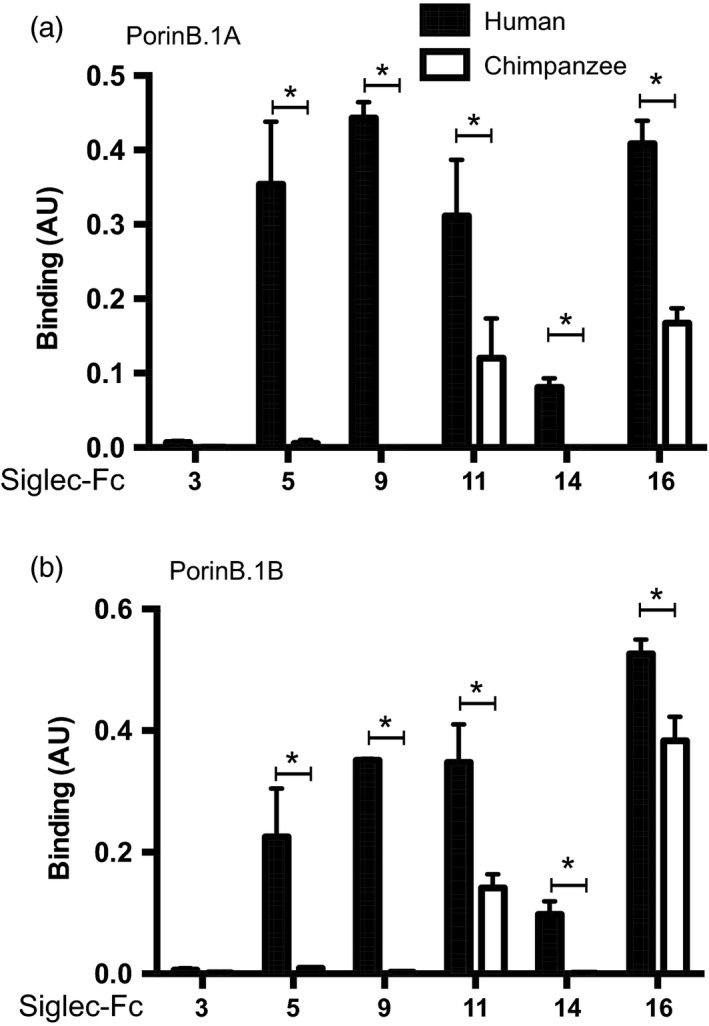
Siglecs bind to the gonococcal porin in a human‐specific manner. Purified PorinB.1A from 15253 (a) and PorinB.1B from F62 (b) were immobilized, and binding of human and chimpanzee Siglec‐Fc proteins was detected with anti‐human IgG‐HRP (Fc specific). Data were corrected for unspecific binding to BSA and represented as mean ± *SD*, *n* = 3, *p*‐values for PorinB.1A and PorinB.1B, respectively, Siglec‐5 *p* = 0.002/0.009, Siglec‐9 *p* < 0.001/<0.001, Siglec‐11 *p* = 0.023/0.006, Siglec‐14 *p* < 0.001/=0.001, and Siglec‐16 *p* < 0.001/=0.006)

In addition to their main sialylated ligands, some Siglecs have been reported to engage in protein–protein interactions (Carlin et al., 2009; Fong et al., 2015). Porin B (PorB) is the major outer membrane protein of *N. gonorrhoeae* and is essential for gonococcal survival. We have shown previously that LOS structure modulates binding of the complement inhibitor C4BP to PorB (Ram et al., 2007). We did not expect the unsialylated glycans expressed by the gonococcal LOS mutants tested in Figure 3c‐g above to directly bind to Siglecs, but instead explored the possibility that alterations in LOS glycan extensions influenced binding of Siglecs to PorB*. *We tested two purified porins, PorB.1A purified from the strain 15253 and PorB.1B derived from the strain F62 (Figure 4). Neither human nor chimpanzee Siglec‐3‐Fc binds to PorB molecules, which is consistent with the finding that binding of human Siglec‐3‐Fc to whole bacteria is solely dependent on LOS sialic acid. PorB.1A and PorB.1B both bound only to human, but not chimpanzee Siglec‐5, Siglec‐9, and Siglec‐14. Both PorB molecules bound to human and chimpanzee Siglec‐11 and Siglec‐16, although binding to chimpanzee Siglecs was much lower. The binding patterns of PorB.1A and PorB.1B were similar, which could indicate a conserved binding region or motif between the two porins. The preference of porins to bind to human Siglecs over chimpanzee homologs strengthens the hypothesis that gonococcal interactions with Siglecs contribute to the human specificity of gonorrhea. It is worth noting that in some instances, whole bacteria, but not the respective purified PorB, bound to chimpanzee Siglecs. For example, F62 bound to cSiglec‐5, cSiglec‐9, and cSiglec‐14, and 15,253 bound to cSiglec‐5 and cSiglec‐14, which suggested that surface molecules other than PorB might engage cSiglecs.

### 3.5 *Neisseria gonorrhoeae* modulates innate immune response via engagement of Siglecs

**Figure 5:**
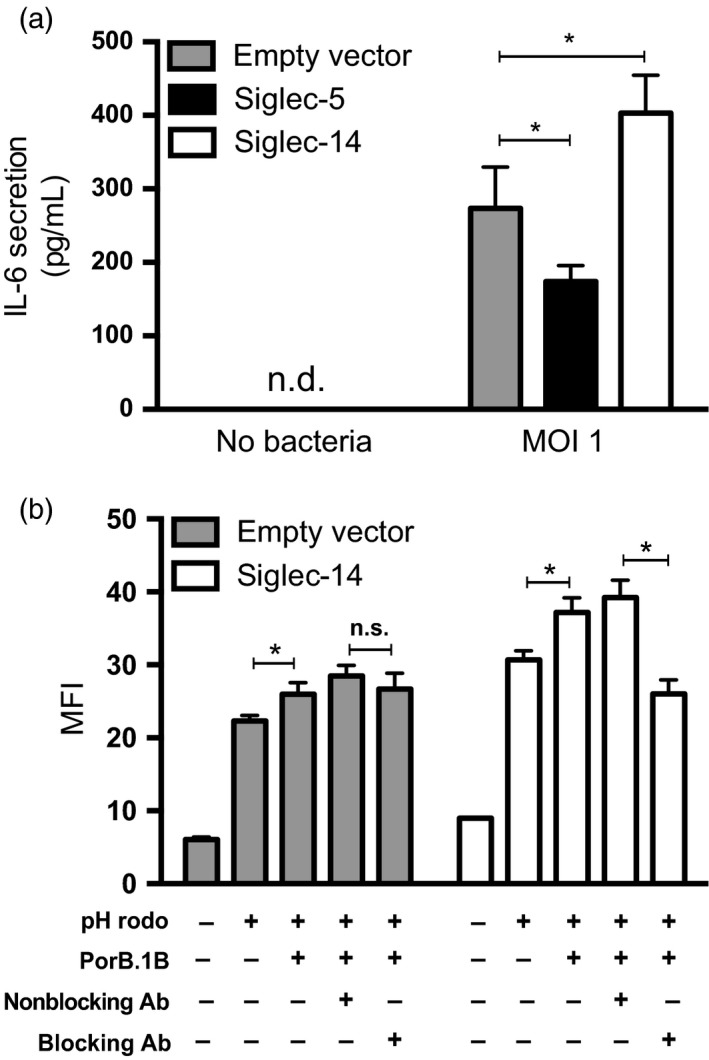
Siglec‐5 and Siglec‐14 modulate pro‐inflammatory response in opposite directions. (a) The secretion of pro‐inflammatory cytokine IL‐6 was measured in THP‐1 expressing either Siglec‐5 or Siglec‐14 after incubation with F62 for 24 hr. (b) Phagocytic activity of THP‐1 cells expressing Siglec‐14 was measured by adding PorB.1B and monitoring the uptake of fluorescent particles (pHrodo). A blocking anti‐Siglec‐14 antibody could reverse the uptake. Data are represented as mean ± SD, *n* = 3

The innate immune system plays an important role in the symptomatology of gonorrhea; the lack of a robust inflammatory response contributes to the lack of symptoms that is commonly seen, especially in women. Monocytes and macrophages are an important source of inflammatory cytokines. Multiple attempts to establish an immortalized cell line expressing Siglec‐11 and Siglec‐16 or human monocytes as model system failed. To investigate whether Siglec interactions with *N. gonorrhoeae* modulate the immune responses, we used a monocytic cell line, THP‐1, which was stably transfected with the paired receptors Siglec‐5 (inhibitory) or Siglec‐14 (activating) (Yamanaka et al., 2009), as a model system. When infected with *N. gonorrhoeae*, THP‐1 cells expressing Siglec‐5 expressed lower level of IL‐6 compared to an empty vector control (*p* = 0.046; Figure 5a). By contrast, expression of the activating Siglec‐14 increased IL‐6 secretion (*p* = 0.043). IL‐6 is a major pro‐inflammatory cytokine, plays an important role during gonococcal infection, and is found in the secretions of infected patients (Ramsey et al., 1995).

In addition to cytokine secretion, phagocytosis is a common response to fight infections. We examined the phagocytic activity of THP‐1 cells expressing Siglec‐14 that were stimulated with purified PorB.1B. Increased phagocytic activity was seen when Siglec‐14‐expressing THP‐1 cells were incubated with PorB.1B (*p* = 0.009). The addition of a Siglec‐14 blocking antibody reversed this effect (*p* = 0.002), suggesting a functional interaction between Siglec‐14 and PorB is a direct physical contact (Figure 5b). These results indicate that gonococcal engagement of inhibitory Siglecs such as Siglec‐5 could contribute to inhibition of pro‐inflammatory responses, as seen in asymptomatic disease. On the other hand, engagement of activating Siglecs, such as Siglec‐14, can contribute to the pro‐inflammatory response that could help clear gonococcal infection.

### 3.6 Genetic polymorphisms in human Siglecs can influence burden of *Neisseria gonorrhoeae*

Similar to Siglec‐16, Siglec‐14 is also not expressed in every individual (Yamanaka et al., 2009). The absence of these activating receptors could contribute to asymptomatic disease and prolonged carriage of gonococci. The rs3865444 polymorphism in Siglec‐3 could potentially also contribute, as it leads to differential expression of two Siglec‐3 isoforms (Malik et al., 2013). The C allele leads to high expression of the full‐length form with a V‐set sialic acid‐binding domain, which can bind to sialylated gonococci, whereas the A allele is associated with lower expression of this isoform. Therefore, the C allele potentially reduces pro‐inflammatory responses by providing gonococci with more exploitable inhibitory Siglec‐3 receptors.

To test whether these genetic variations in human Siglecs are associated with gonococcal infections, we genotyped a human cohort with a high burden of gonorrhea. A cross‐sectional study of Namibian pastoralists has shown that over 64% of the tested sexually active adult population is infected with *N. gonorrhoeae* with a higher prevalence in females (72%) compared to males (57%; Hazel et al., 2014). In this rural population where partner concurrency is normal for men and women, gonorrhea exposure is high but treatment is erratically sought.

In this cohort, we found that infected females express the Siglec‐16 receptor less frequently with an allele frequency of the SIGLEC16 with 0.155 compared to uninfected women with 0.213 (Supporting Information Table S1). We further found trends showing higher expression of activating Siglec‐14 receptor expressed in the uninfected population with an allele frequency of 0.729 compared to 0.693 in the infected population. If statistically significant, this trend could support our hypothesis that activating Siglec‐14 is protective. We saw a similar trend when females and males were analyzed separately. The pathology of the gonococcal infection is very different between males and females, and therefore, it is important to look at the two populations separately. There were no significant differences between the A and C alleles in Siglec‐3 among infected and uninfected persons.

**Figure 6:**
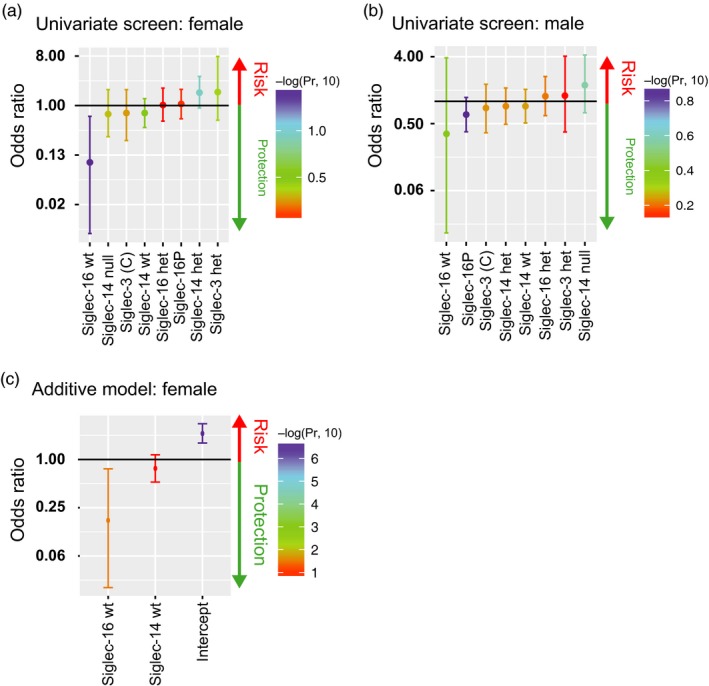
Activating Siglecs are protective. Univariate screen of females (a) and males (b). Additive model of females, who are homozygous for SIGLEC16 and Siglec‐14 wt (c)

To test the statistical significance of these observations, we used a univariate logistic regression screen within the female population and found a significant protective association, odds ratio of 0.092 (*p* = 0.034; 95% confidence interval (CI) = 0.005–0.636), for women with the homozygous genotype for Siglec‐16 (Figure 6a, Supporting Information Table S2). The same analysis showed no significant protection or risk for the different genotypes in males (Figure 6b). In an additive model of homozygous Siglec‐14 wt and Siglec‐16 wt, Siglec‐16 again showed again significant protection with an odds ratio of 0.089 (*p* = 0.032; CI = 0.004–0.620); however, Siglec‐14 alone was not significant, with an odds ratio of 0.713 (*p* = 0.280; CI = 0.384–1.318; Figure 6c, Supporting Information Table S2).

It is possible that the greater significance of the Siglec‐16 genotype may result because Siglec‐16 is expressed on macrophages and also on the cervical epithelium, which is the main site of infection. This additional site of expression could enhance contact with gonococci and thus drive selection for the protective Siglec‐16 genotype. It is worth noting that immortalized cervical epithelial cells also release pro‐inflammatory cytokines following stimulation with *N. gonorrhoeae* (Fichorova, Desai, Gibson, & Genco, 2001).

## 4 DISCUSSION

*Neisseria gonorrhoeae* has evolved a variety of strategies to infect the human host and exploit its immune system (Edwards & Apicella, 2004). In this study, we present a new mechanism whereby *N. gonorrhoeae *engages human Siglec receptors to suppress pro‐inflammatory signaling. Natural infection with *N. gonorrhoeae* is restricted to humans. In the 1970s, a urethral model of gonorrhea in male chimpanzees (*Pan troglodytes*) was established (Arko, 1989). Infection occurred by some, but not all strains of *N. gonorrhoeae* (Arko, Duncan, Brown, Peacock, & Tomizawa, 1976; Kraus, Brown, & Arko, 1975). Similar to humans, localized urethral infection in chimpanzees lasted from 3 to 6 weeks (Lucas, Chandler, Martin, & Schmale, 1971) and male‐to‐female transmission among sexually active cage mates was documented (Brown, Lucas, & Kuhn, 1972). Several reasons for the natural host restriction of gonococcal infection have been elucidated, including specificity for human CEACAMs (Pils, Gerrard, Meyer, & Hauck, 2008; Sadarangani, Pollard, & Gray‐Owen, 2011; Sintsova et al., 2015; Voges, Bachmann, Kammerer, Gophna, & Hauck, 2010), the ability to utilize transferrin as an iron source from humans and only certain non‐human primates (Gray‐Owen & Schryvers, 1993) and the inability to evade non‐human complement because of its selective binding to the human complement inhibitors, factor H and C4b‐binding protein (C4BP) (Ngampasutadol et al., 2005, 2008). Our results show that *N. gonorrhoeae* binds preferentially to human Siglecs, which may be another reason for its human specificity.

**Figure 7:**
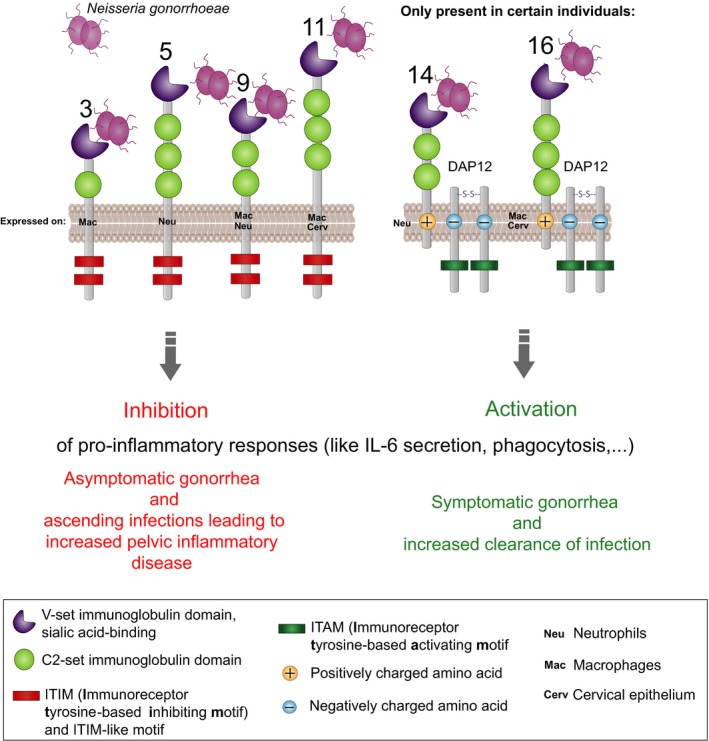
Possible mechanism of Siglec gonococci interactions. *Neisseria gonorrhoeae* engages human immunoregulatory Siglec receptors. Upon binding, the inhibitory receptors Siglec‐3, Siglec‐5, Siglec‐9 and, Siglec‐11 suppress the pro‐inflammatory response, which could contribute to decreased clearance of infection and overall appearance of an asymptomatic infection. The engagement of activating Siglec receptors, such as Siglec‐14 and Siglec‐16, leads to an activation of the pro‐inflammatory signaling cascade. This can contribute to a better clearance of a symptomatic infection. However, these activating receptors are expressed only on certain individuals. The presence of these polymorphisms could contribute to different ability of clearing a gonococcal infection among individuals

Apart from these evolutionary considerations, the data presented in this work suggest that interaction of *N. gonorrhoeae *with Siglecs plays a role in the innate immune response during infection (see summary in Figure 7). Binding to the inhibitory receptors Siglec‐3, Siglec‐5, Siglec‐9, and Siglec‐11 suppresses the pro‐inflammatory response, which could contribute to decreased clearance of infection and contribute to the relative paucity of symptoms. The engagement of activating Siglec receptors, such as Siglec‐14 and Siglec‐16, leads to an activation of the pro‐inflammatory signaling cascade that facilitates clearance of gonococcal infection. However, these activating receptors are not present on all individuals, which may account, at least in part, for differences across individuals in their ability to clear gonococcal infections.

The role of pro‐inflammatory responses in gonococcal pathogenesis remains controversial (Criss & Seifert, 2012; Virji, 2009). While activating receptors such as Siglec‐14 and Siglec‐16 may contribute to a pro‐inflammatory response following infection facilitate clearance of infection, *N. gonorrhoeae* have developed numerous mechanisms to survive within neutrophils that are abundant at the site of infection upon pro‐inflammatory stimuli, which could form a “safe haven” for bacteria (Criss & Seifert, 2012). Multiple factors, such as sex and site of infection, play a role in the effectiveness of a pro‐inflammatory stimulus. The genetic analysis of the Namibian pastoralists shows that the presence of activating Siglec‐16 is correlated with a lower burden of gonorrhea in females. Siglec‐16 is expressed by cervical epithelium in addition to immune cells, thus making it an activating Siglec that gonococci are likely to encounter in the early stages of infection. This could explain why Siglec‐16 has stronger effect on the gonococcal burden compared to the activating Siglec‐14, which is only expressed on neutrophils. It is intriguing that unsialylated *N. gonorrhoeae* bind better to Siglec‐16 than sialylated bacteria. Ketterer et al. (2016) recently showed that sialidases are present in the female genital tract in amounts sufficient to desialylate gonococcal LOS and enhance infectivity in men, where unsialylated gonococci can engage the asialoglycoprotein receptor (ASGP‐R; Harvey et al., 2001). The ability of Siglec‐16 to respond to unsialylated gonococci may represent an evolutionary mechanism that permits the host to adapt to gonococci whose surface has been modified by sialidases elaborated by cohabiting microbial flora (Ketterer et al., 2016). Ascending gonococcal infections can also cause pelvic inflammatory disease, a major cause of infertility (Gradison, 2012) (Reekie et al., 2017). Whether interactions between *N. gonorrhoeae* and Siglecs contribute to pelvic inflammatory disease and infertility merits further study. Any impact on fertility would be a powerful driver of selection for *SIGLEC* gene polymorphisms.

On a more general note, we and others have reported how the human specificity of certain infectious diseases such as *Salmonella typhi‐*mediated typhoid fever and *Plasmodium falciparum* malaria might be explained by the human‐universal genetic loss of the common mammalian sialic acid Neu5Gc (Deng et al., 2014; Martin, Rayner, Gagneux, Barnwell, & Varki, 2005). These organisms have evolved modification of certain binding proteins to preferentially recognize the precursor sialic acid Neu5Ac, which accumulates in excess in humans. While no pathogens are known to synthesize Neu5Gc, other human pathogens have independently evolved convergent ways to coat themselves with Neu5Ac containing glycans, and thereby evade the innate immune response via multiple mechanisms, including the engagement of inhibitory Siglecs on innate immune cells (Chang & Nizet, 2014; Varki & Gagneux, 2012; Vimr, Kalivoda, Deszo, & Steenbergen, 2004). Two such documented examples include human‐specific pathogens group B streptococcus which engages Siglec‐9 on neutrophils and Siglec‐5 on the amniotic epithelium (Ali et al., 2014; Carlin et al., 2007) and *E. coli* K‐1 which engages Siglec‐11 on brain microglia, likely suppressing their microbicidal activity during the process of causing meningitis (Schwarz et al., 2017). Here, we report a different evolutionary strategy for human specificity, employed by a bacterium, which largely bypasses the sialic acid‐binding properties of Siglecs and instead engages them directly via protein–protein interactions. The other pathogen in the genus Neisseria, *N. meningitidis,* also engages Siglec‐1 and Siglec‐5 which results in increased bacterial phagocytosis; however, the interaction occurred exclusively through sialylated lacto‐*N*‐neotetraose LOS (Jones, Virji, & Crocker, 2003).

## CONFLICT OF INTEREST

None declared.

## AUTHOR CONTRIBUTIONS

CSL, SR, and AV designed the research study. CSL, JJF, FS, and AS conducted the experiments. CSL, AH, BJK, NV, and NEL analyzed data. AH and PM provided reagents. CSL, SR, and AV wrote the manuscript.

## DATA ACCESSIBILITY

Data for this study are available at Dryad Digital Repository: https://doi.org/10.5061/dryad.4b2158p .
