# Supplementary figures and images for: Evolution of the exclusively human pathogen Neisseria gonorrhoeae: Human‐specific engagement of immunoregulatory Siglecs
Source: Evol Appl. 2019 Jan 3;12(2):337–49. doi: 10.1111/eva.12744 (PMC6346652; doi:10.1111/eva.12744)

A

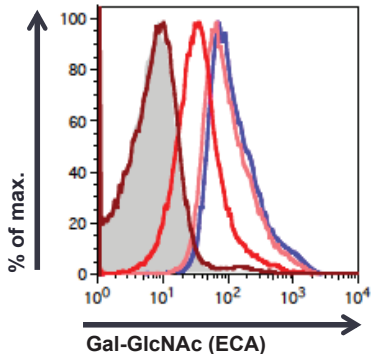

wt + 0  $\mu$ M CMP-Neu5Ac  
wt + 0  $\mu$ M CMP-Neu5Ac  
+ 200  $\mu$ M Lactose  
wt + 30  $\mu$ M CMP-Neu5Ac  
wt + 3  $\mu$ M CMP-Neu5Ac  
wt + 0.3  $\mu$ M CMP-Neu5Ac

Supplement: Supplementary file 1 [file EVA-12-337-s001.pdf]

**A**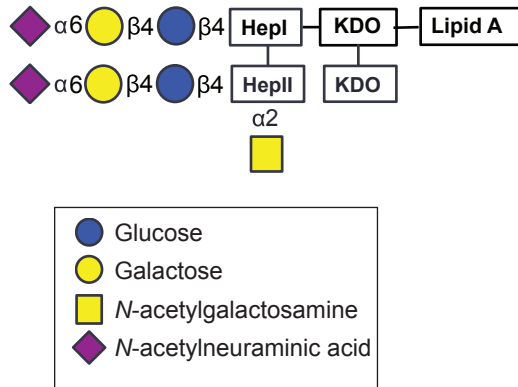**B**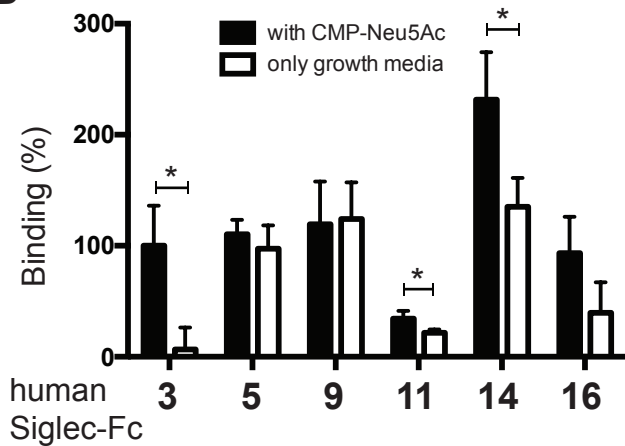

Supplement: Supplementary file 6 [file EVA-12-337-s006.pdf]
