# Supplementary material for: Evolution of the exclusively human pathogen Neisseria gonorrhoeae: Human‐specific engagement of immunoregulatory Siglecs
Source: Evol Appl. 2019 Jan 3;12(2):337–49. doi: 10.1111/eva.12744 (PMC6346652; doi:10.1111/eva.12744)
Supplement: Supplementary file 2 [file EVA-12-337-s002.pdf]

```
                    V-set
Human Siglec-3       MPLLLLLPLLWAGALAMDPNFWLQVQESVTVQEGLCVLVPCTFFHPIPYYDKNSPVHGYW  60
Chimpanzee Siglec-3  MPLLLLLPLLWAGALAMDPKIRLQVQESVTVQEGLCVLVPCTFFHPIPYYDKNSPVHGYW  60
                     ******************* :: **************************************

                     FREGAIISRDSPVATNKLDQEVQEETQGRFRLLGDPSRNNCSLSIVDARRRDNGSYFFRM  120
                     FREGPIVSGDSPVATNKPDQEVQEETQGRFRLLGDLSRNNCSLSIVDARRRDNGSYFFRM  120
                     **** *:* ******** ***************** ************************
                                                  C2 type
                     ERGSTKYSYKSPQLSVHVTDLTHRPKILIPGTLEPGHSKNLTCSVSWACEQGTPPIFSWL  180
                     ERGSTKYSYKSPQLSVHVTDLTHRPKILIPGALDPGHSKNLTCSVSWACEQGTPPIFSWL  180
                     *******************************:*:**************************

                     SAAPTSLGPRTTHSSVLIITPRPQDHGTNLTCQVKFAGAGVTTERTIQ  228
                     SAAPTSLGPRTTHSSVLIITPRPQDHGTNLTCQVKFAGAGVTTERTIQ  228
                     ************************************************
```

Sequence identity: 96% (218/228)
