# Supplementary material for: Evolution of the exclusively human pathogen Neisseria gonorrhoeae: Human‐specific engagement of immunoregulatory Siglecs
Source: Evol Appl. 2019 Jan 3;12(2):337–49. doi: 10.1111/eva.12744 (PMC6346652; doi:10.1111/eva.12744)
Supplement: Supplementary file 3 [file EVA-12-337-s003.pdf]

```
                              V-set
Human Siglec-5          MLPLLLLPLLWGGSLQEKPVYELQVQKSVTVQEGLCVLVPCSFSYPWRSWYSSPPLYVYW   60
Chimpanzee Siglec-5     MLPLLLLPLLWGGSLQEEPGYELQVQKSVTVQEGLCVLVSCSFSYPWRSWYSSPRLYIYW   60
                        *****************:* ******************* ************** **:**

                        FRDGEIPYYAEVVATNNPDRRVKPETQGRFRLLGDVQKKNCSLSIGDARMEDTGSYFFRV   120
                        FRDGESPYYAEAVATNNLDGRVKPGTRGRFRLLGDVQKKNCSLSIGDARMEDTGSYFFHV   120
                        ***** *****.***** * **** *:*******************************:*

                                             C2 type
                        ERGRDVKYSYQQNKLNLEVTALIEKPDIHFLEPLESGRPTRLSCSLPGSCEAGPPLTFSW   180
                        ERGRDVKHSYQQNKLNLEVTALIEKPDIHFLEPLESGRPTRLSCSLPGSCEAGRPLTFSW   180
                        *******:********************************************* ******

                                                                             C2 type
                        TGNALSPLDPETTRSSELTLTPRPEDHGTNLTCQMKRQGAQVTTERTVQLNVSYAPQTIT   240
                        TGNALSPLDPETTRSSELTLTPRPEDHGTNLTCRVKRQGAQVTTERTVQLNVSYAPQTIT   240
                        *********************************::*************************

                        IFRNGIALEILQNTSYLPVLEGQALRLLCDAPSNPPAHLSWFQGSPALNATPISNTGILE   300
                        IFRNGIALEILQNTSYLPVLEGQALRLLCDAPSNPPAHLSWFQGSPALNATPISNTGILE   300
                        ************************************************************

                        LRRVRSAEEGGFTCRAQHPLGFLQIFLNLSVYSLPQLLGPSCSWEAEGLHCRCSFRARPA   360
                        LRRVRSAEEGSFTCRAQHPLGFLQIFLNLSVYSLPQLLGPSCSWEAEGLHCSCSFRARPA   360
                        **********.**************************************** ********

                        PSLCWRLEEKPLEGNSSQGSFKVNSSSAGPWANSSLILHGGLSSDLKVSCKAWNIYGSQS   420
                        PSLCWWLGEKPLEGNSSQGSFKVNSSSAGPWANSSLILHGGLSSDLKVSCKAWNSYGSQS   420
                        ***** * ********************************************** *****

                        GSVLLLQGRSNLGTGVV    437
                        GSVLLLQGRSNLGTGVV    437
                        *****************
```

Sequence identity: 97% (426/437)
