# Supplementary material for: Evolution of the exclusively human pathogen Neisseria gonorrhoeae: Human‐specific engagement of immunoregulatory Siglecs
Source: Evol Appl. 2019 Jan 3;12(2):337–49. doi: 10.1111/eva.12744 (PMC6346652; doi:10.1111/eva.12744)
Supplement: Supplementary file 4 [file EVA-12-337-s004.pdf]

```
V-set
Human Siglec-9       MLLLLLPLLWGRERAEGQTSKLLTMQSSVTVQEGLCVHVPCSFSYPSHGWIYPGPVVHGY  60
Chimpanzee Siglec-9  MLLLLLPLLWGRERAEGQTSNLLTMQSSVMVQEGLCVHVPCSFSYPSRGWIYPGPVVHGY  60
                     ********************:******** *****************:************

                     WFREGANTDQDAPVATNNPARAVWEETRDRFHLLGDPHTKNCTLSIRDARRSDAGRYFFR  120
                     WFREGANTDQDAPVATNNPARAVREETRDRFHLLGDPHTKNCTLSIRDARRSDAGRYFFR  120
                     *********************** ************************************

C2 type
                     MEKGSIKWNYKHHRLSVNVTALTHRPNILIPGTLESGCPQNLTCSVPWACEQGTPPMISW  180
                     VETGNIKWNYKHHRLSVNVTALTHRPNILIPGTLESGCPQNLTCSVPWACEQGTPPMISW  180
                     :*.*.*******************************************************

C2 type
                     IGTSVSPLDPSTTRSSVLTLIPQPQDHGTSLTCQVTFPGASVTTNKTVHLNVSYPPQNLT  240
                     IGTSVSPLDPSTTHSSVLTLIPQPQDHGTSLTCQVTFPGASVTTNKTVHLNVSYPPQNLT  240
                     *************:**********************************************

                     MTVFQGDGTVSTVLGNGSSLSLPEGQSLRLVCAVDAVDSNPPARLSLSWRGLTLCPSQPS  300
                     MTVFQGDGTVSTVLGNGSSLSLPEGQSLRLVCAVDAVDSNPPARLSLSWRGLTLCPSQPS  300
                     ************************************************************

                     NPGVLELPWVHLRDAAEFTCRAQNPLGSQQVYLNVS  336
                     NPGVLELPWVHLRDEDEFTCRAQNPLGSQQVSLNVS  336
                     **************  *************** ****
```

Sequence identity: 97% (325/336)
