# Supplementary material for: Evolution of the exclusively human pathogen Neisseria gonorrhoeae: Human‐specific engagement of immunoregulatory Siglecs
Source: Evol Appl. 2019 Jan 3;12(2):337–49. doi: 10.1111/eva.12744 (PMC6346652; doi:10.1111/eva.12744)
Supplement: Supplementary file 5 [file EVA-12-337-s005.pdf]

```
                                         V-set
Human Siglec-14       MLPLLLLPLLWGGSLQEKPVYELQVQKSVTVQEGLCVLVPCSFSYPWRSWYSSPPLYVYW 60
Chimpanzee Siglec-14  MLPLLLLPLLWGGSLQEEPGYELQVQKSVTVQEGLCVLVSCSFSYPWRSWYSSPPLYVYW 60
                      *****************:* ******************* ********************

                      FRDGEIPYYAEVVATNNPDRRVKPETQGRFRLLGDVQKKNCSLSIGDARMEDTGSYFFRV 120
                      FRDGESPYYAEAVATNNLDGRVKPGTRGRFRLLGDVQKKNCSLSIGDARMEDTGSYFFHV 120
                      ***** *****.***** * **** *:*******************************:*

                                                   C2 type
                      ERGRDVKYSYQQNKLNLEVTALIEKPDIHFLEPLESGRPTRLSCSLPGSCEAGPPLTFSW 180
                      ERGRDVKHSYQQNKLNLEVTALIEKPDIHFLEPLESGHPTRLSCSLPGSCEAGRPLTFSW 180
                      *******:*****************************:*************** ******

                                                                         C2 type
                      TGNALSPLDPETTRSSELTLTPRPEDHGTNLTCQVKRQGAQVTTERTVQLNVSYAPQNLA 240
                      TGNALSPLDPETTRSSELTLTPRPEDHGTNLTCQVKRQGAQVTTERTVQLNVSYAPQNLA 240
                      ************************************************************

                      ISIFFRNGTGTALRILSNGMSVPIQEGQSLFLACTVDSNPPASLSWFREGKALNPSQTSM 300
                      ISIFFRNGTGTALRILSNGMSVPIQEGQSLFLACTVDSNPPASLSWFREGKALNPSQTSM 300
                      ************************************************************

                      SGTLELPNIGAREGGEFTCRVQHPLGSQHLS     331
                      SGTLELPNIGAREGGEFTCRVQHPLGSQHLS     331
                      *******************************
```

Sequence identity: 96% (318/331)
