# Supplementary material for: Evolution of the exclusively human pathogen Neisseria gonorrhoeae: Human‐specific engagement of immunoregulatory Siglecs
Source: Evol Appl. 2019 Jan 3;12(2):337–49. doi: 10.1111/eva.12744 (PMC6346652; doi:10.1111/eva.12744)
Supplement: Supplementary file 7 [file EVA-12-337-s007.pdf]

Siglec-5/Siglec-14		wt	het	null	total	Allele Frequency wt	Allele Frequency null
all subjects	uninfected	81	48	15	**144**	0.729	0.271
	infected	129	98	30	**257**	0.693	0.307
female							
	uninfected	30	16	7	**53**	0.717	0.283
	infected	65	59	13	**137**	0.690	0.310
male							
	uninfected	51	32	8	**91**	0.736	0.264
	infected	64	39	17	**120**	0.696	0.304

Siglec-16/Siglec-16P		Siglec-16P	het	Siglec-16	total	Siglec-16P	Siglec-16
all subjects	uninfected	98	39	6	**143**	0.822	0.178
	infected	164	74	2	**240**	0.838	0.163
female							
	uninfected	35	15	4	**54**	0.787	0.213
	infected	92	39	1	**132**	0.845	0.155
male							
	uninfected	63	24	2	**89**	0.843	0.157
	infected	72	35	1	**108**	0.829	0.171

Siglec-3 rs3865444 (C/A)		C	het	A	total	C	A
all subjects	uninfected	140	8	1	**149**	0.966	0.034
	infected	247	21	1	**269**	0.957	0.043
female							
	uninfected	54	3	0	**57**	0.974	0.026
	infected	134	13	0	**148**	0.956	0.044
male							
	uninfected	86	5	1	**92**	0.962	0.038
	infected	113	8	1	**122**	0.959	0.041
