# Supplementary material for: Evolution of the exclusively human pathogen Neisseria gonorrhoeae: Human‐specific engagement of immunoregulatory Siglecs
Source: Evol Appl. 2019 Jan 3;12(2):337–49. doi: 10.1111/eva.12744 (PMC6346652; doi:10.1111/eva.12744)
Supplement: Supplementary file 8 [file EVA-12-337-s008.pdf]

Univeriate Screen			Odds Ratio	P-value	95% CI
	female				
		Siglec-16 wt	0.092	0.034	(0.005-0.636)
		Siglec-14 null	0.700	0.472	(0.271-1.951)
		Siglec-3 (C)	0.730	0.555	(0.231-1.951)
		Siglec-14 wt	0.731	0.308	(0.398-1.337))
		Siglec-16 het	1.021	0.952	(0.519-2.081)
		Siglec-16P	1.069	0.831	(0.575-1.969)
		Siglec-14 het	1.724	0.106	(0.904-3.409)
		Siglec-3 het	1.758	0.392	(0.541-7.881)
	male				
		Siglec-16 wt	0.365	0.413	(0.017-3.859)
		Siglec-16P	0.664	0.131	(0.388-1.126)
		Siglec-3 (C)	0.813	0.588	(0.377-1.698)
		Siglec-14 het	0.860	0.598	(0.490-1.513)
		Siglec-14 wt	0.861	0.571	(0.511-1.447)
		Siglec-16 het	1.168	0.615	(0.017-3.859)
		Siglec-3 het	1.194	0.763	(0.386-4.058)
		Siglec-14 null	1.653	0.265	(0.702-4.208)
Additive Model					
	female				
		Siglec-16 wt	0.089	0.032	(0.004-0.620)
		Siglec-14 wt	0.713	0.280	(0.384-1.318)
		Intercept	3.201	1.39E-07	(2.106-5.025)
	male				
		Intercept	1.722	0.009	(1.149-2.620)
		Siglec-16P	0.664	0.131	(0.388-1.126)
